# Distinct DNA hydroxymethylation landscape and mitochondrial DNA rearrangement signatures induced by single- and multi-fraction alpha particle radiation in lung fibroblasts

**DOI:** 10.1093/eep/dvag020

**Published:** 2026-07-23

**Authors:** Marilyn N Vera-Chang, John M Danforth, Marilyne Stuart, Marjorie Brand, Aaron A Goodarzi, Richard B Richardson

**Affiliations:** Radiobiology and Health Branch, Chalk River Laboratories, Canadian Nuclear Laboratories, Chalk River, ON, K0J 1J0, Canada; Robson DNA Science Centre, Departments of Biochemistry and Molecular Biology and Oncology, Charbonneau Cancer Institute, Cumming School of Medicine, University of Calgary, Calgary, AB, T2N 1N4, Canada; Environment and Waste Technologies Branch, Chalk River Laboratories, Canadian Nuclear Laboratories, Chalk River, ON, K0J 1J0, Canada; Ottawa Hospital Research Institute, Ottawa, ON, K1H 8L6, Canada; Department of Cellular and Molecular Medicine, University of Ottawa, Ottawa, ON, K1H 8L6, Canada; Robson DNA Science Centre, Departments of Biochemistry and Molecular Biology and Oncology, Charbonneau Cancer Institute, Cumming School of Medicine, University of Calgary, Calgary, AB, T2N 1N4, Canada; Radiobiology and Health Branch, Chalk River Laboratories, Canadian Nuclear Laboratories, Chalk River, ON, K0J 1J0, Canada; McGill Medical Physics Unit, Cedars Cancer Centre–Glen Site, Montreal, QC, H4A 3J1, Canada

**Keywords:** aging, alpha particles, hydroxymethylome, lung, mitochondria, radiation, radon

## Abstract

Alpha (α)-radiation is a genotoxic and epigenotoxic agent capable of increasing the risk of cancer and other diseases. DNA hydroxymethylation (DNAhm) can modulate gene expression and is considered an under-researched epigenetic event in ionizing radiation studies. Here, the genome-wide DNAhm profile was examined in 64 α-irradiated human embryonic lung fibroblast samples from our 2023 methylome study. These cells were exposed to seven doses (*N* = 4 per dose) of americium-241 α-particles ranging from 2 to 2200 mGy, using either single-fraction (SF) or multi-fraction (MF) exposure regimens. We report that SF and MF α-irradiation primarily increased DNAhm levels, with a greater number of alterations observed following MF exposure, particularly in gene body regions. Pathway analysis of genes with increased DNAhm levels due to MF exposure suggested disruption of inflammatory responses and other cellular pathways. We also detected DNAhm changes in key genes encoding enzymes involved in DNA methylation (DNAm) and demethylation processes, which support our 2023 DNAm findings. Similar to DNAm, MF α-irradiation induced a greater number of DNAhm changes in aging-associated genes. Compared with equivalent SF doses, MF α-irradiation resulted in more mitochondrial damage. These findings demonstrate that even at comparable doses, SF and MF α-radiation induce radically different effects on the DNAhm and mitochondrial DNA. The DNAhm changes help explain the effects observed in our 2023 methylome study. Altogether, this study supports the idea that environmental radiation exposure regimens are an important consideration when assessing DNAm and DNAhm biomarkers and the potential health effects of α-radiation.

## Graphical Abstract

**Graphical Abstract:**
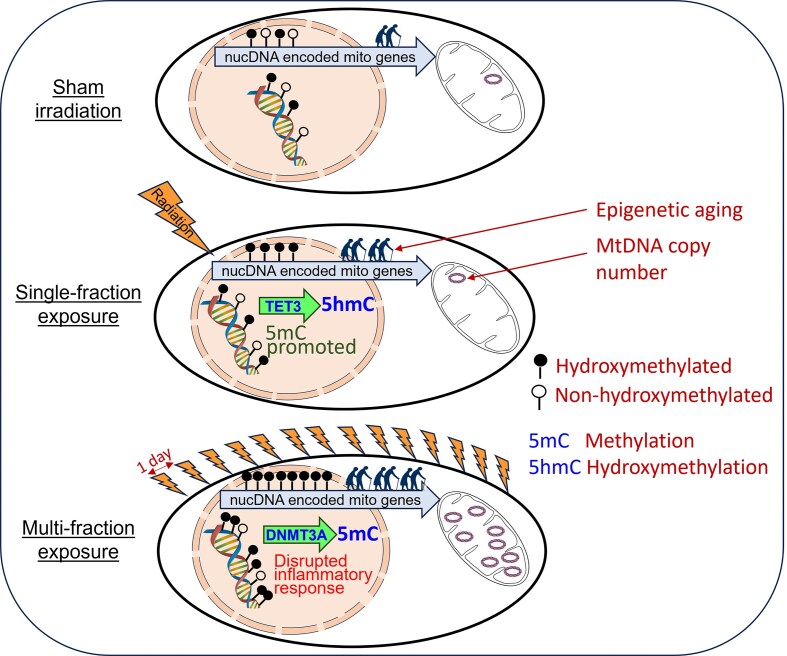

## Background

Soon after Wilhelm Röntgen discovered X-rays in 1895, it was established that ionizing radiation (IR) posed a health risk to humans [1]. The effects of IR exposure are largely dependent on the dose, dose rate, and radiation quality [2–4]. Due to the increasing presence of the alpha (α-)emitter radon-222 (^222^Rn) at unsafe levels in enclosed spaces, as a result of modern construction and heat-conserving practices [5–7], α-particle radiation is the most significant natural source of IR to which humans are exposed [8]. The radioactive noble gas ^222^Rn is generated from the decay chain of geogenic uranium and radium present in rocks and soil [9]. ^222^Rn itself emits four α-particles before reaching its stable end product, lead-206 [10]. Upon inhalation of α-radiation-emitting radionuclides, α-particles can induce genotoxic effects in lung tissues, which increases the risk of cancer [9].

It is now evident that IR induces not only genetic damage but also epigenetic modifications [11], which are alterations in gene expression, independent of changes in the original DNA sequence. Epigenetic events play a key role in modulating gene expression during cellular development and differentiation [12]. They are also known to be highly responsive to various stimuli, including environmental challenges such as pro-oxidant IR. Some epigenetic responses and their biological outcomes are partially reversible, but others persist, even for decades and generations [13].

Here, we investigated the landscape of DNA hydroxymethylation (DNAhm), an understudied epigenetic mark, following exposure to α-radiation using different modalities of exposure. DNAhm occurs during the active DNA demethylation process, leading to the oxidation of 5-methylcytosine (5mC), also known as the “fifth base” of DNA. This enzymatic reaction is catalyzed by the ten-eleven translocation (TET) Fe^2+^ 2-oxoglutarate dioxygenase family of enzymes, consequently forming 5-hydroxymethylcytosine (5hmC), or the “sixth base” of DNA [14]. Five-hydroxymethylcytosine was previously considered merely an intermediate step in the DNA demethylation pathway; however, given its stability, 5hmC, similar to 5mC, plays a significant role in gene regulation [11]. Global 5hmC levels across the human genome are tissue-specific [14] and approximately ten-fold lower than 5mC levels [15]. Multi-tissue studies have revealed that 5hmC is preferentially enriched in gene body regions, promoters, and enhancers of transcriptionally active genes [14, 16–19].

Due to their role in modulating transcriptional patterns, epigenetic mechanisms are regarded as the cardinal features of cellular aging, as they change throughout an individual’s lifetime in a cell-specific manner. Aging is a complex natural event characterized by a progressive decline of biological processes and cellular functions [20], and is a major risk factor for many diseases such as cancer [21], cardiovascular pathologies [22], neurodegeneration [23], and chronic inflammatory diseases [24, 25]. Given the reversible nature of epigenetic marks and their capacity to be influenced by many different environmental and genetic factors, IR can potentially affect an individual’s biological age through epigenetic events [26]. To date, one of the most studied aging-related epigenetic mechanisms is DNA methylation (DNAm) [27]. Considering the significant enrichment of 5hmC in the central nervous system [28], prevailing research conducted thus far has focused on elucidating the role of 5hmC in the aging process of the brain. Consequently, current knowledge of 5hmC dynamics in aging remains scarce in non-brain tissues, including lung fibroblasts.

It is widely recognized that mitochondria serve as the cell’s main energy source; however, it is less known that mitochondria provide key intermediate metabolites necessary for epigenetic regulation [29]. Conversely, epigenetic mechanisms can also modulate the mitochondrial genome, with 5mC being the primary focus in most studies. Given these roles, assessing the effects of IR on 5hmC levels of the mitochondrial DNA (mtDNA) will bring us a step closer to a better understanding of the dynamics between epigenetic effects and mitochondrial processes in response to pro-oxidant IR exposures.

Here, we surveyed global 5hmC levels in normal, three-month-old derived human lung fibroblast cells irradiated with α-particles using two exposure regimens, namely single-fraction (SF) and multi-fraction (MF) delivery methods. The SF exposure (total IR dose delivered in a single day) enables us to examine hypothetically the effects of acute, high-dose α-radiation exposure resulting from a dirty bomb detonation or a nuclear incident. In contrast, the MF irradiation (total IR dose delivered as 14 equal daily fractions administered at 24-h intervals) simulates repetitive low-dose residential exposure to the ubiquitous inert gas ^222^Rn. We used seven doses ranging from 2 to 2,200 mGy to reflect the variability in worldwide human exposure to the α-emitter ^222^Rn. For the assessment of mitochondrial endpoints, an additional experiment was performed using doses ranging from 7 to 14,000 mGy to evaluate responses at higher radiation doses. To our knowledge, this is the first study to report on the profile of 5hmC, an important and understudied epigenetic modification, following exposure to α-radiation. Our results revealed a dose-dependent increase in DNAhm levels in both SF and MF α-irradiations. We identified 5hmC changes in key genes encoding enzymes associated with DNAm and DNAhm-dependent demethylation processes, which explains the results from our previous study regarding the methylome of SF and MF α-irradiated fibroblasts [25]. Lastly, here we showed that the mitochondria experienced more damage upon MF α-irradiation compared with SF exposure.

## Results

### Distinct dose-response effect of SF and MF α-irradiations on the 5hmC levels of lung fibroblasts

Distribution of 5hmC sites across the genome of sham- and α-irradiated human WI-38 cells, a normal fibroblast cell line derived from the lung tissue of a 3-month-old female, was assessed by immunoprecipitation followed by deep sequencing of the genomic regions enriched for hydroxymethylated DNA (hydroxymethylated DNA immunoprecipitation coupled with sequencing, hMeDIP-seq). Seven human-relevant doses of α-particles, ranging from a very low dose of 2.0 mGy to a high dose of 2200 mGy, were administered to the cells using either a SF or a MF exposure regimen. Fibroblasts irradiated to SF and MF exposures are referred to by a superscript denoting the total delivered dose in mGy as follows: SF^2^, SF^11^, SF^23^, SF^110^, SF^220^, SF^1100^, SF^2200^, MF^2^, MF^11^, MF^23^, MF^110^, MF^220^, MF^1100^, and MF^2200^. The number of differentially hydroxymethylated regions (DhMRs) detected between the controls and each of the irradiated groups is provided in Supplementary file1, Table S1.

**Figure 1:**
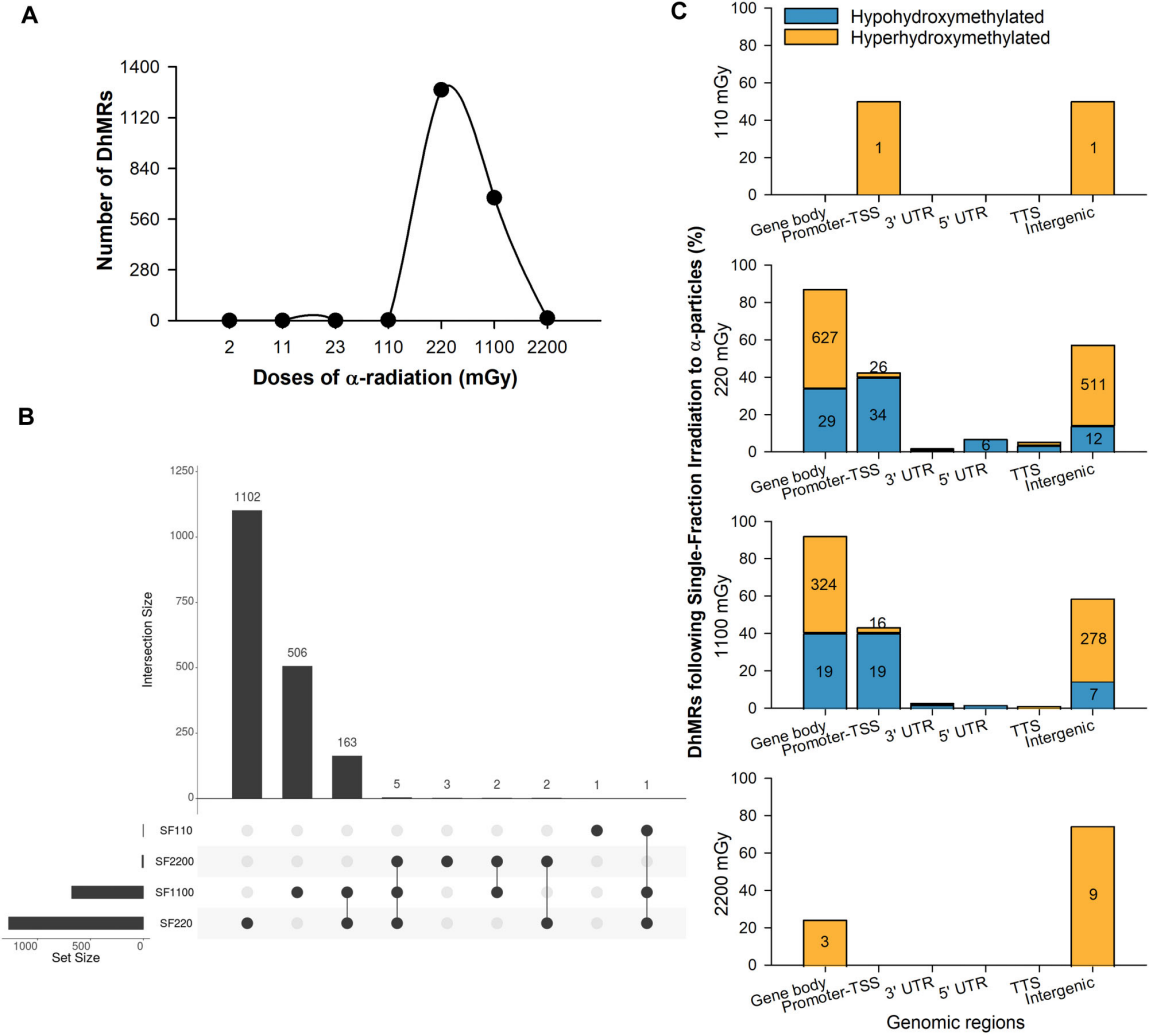
Differentially hydroxymethylated regions (DhMRs) in lung fibroblasts following single-fraction (SF) α-irradiation. (A) Total number of DhMRs across seven americium-241 α-particle doses identified using the Bioconductor MEDIPS package. (B) UpSet plot displaying the number of identified DhMRs in the fibroblasts across the different SF α-irradiation doses. (C) Percentages of the genomic regions found to be hypo- and hyper-hydroxymethylated in each irradiated group. The integer inside each bar represents the number of DhMRs. Each irradiation group includes four biological replicates per dose, independently irradiated and assessed. TSS, transcription start site; TTS, transcription termination site; 3’ UTR, 3’ untranslated region; 5’ UTR, 5’ untranslated region.

Differential analysis between the SF-irradiated fibroblasts and the sham-irradiated cells revealed a non-monotonic dose-response with the following DhMRs’ distribution, 2; 1 273; 677; and 12 for SF^110^, SF^220^, SF^1100^, and SF^2200^, respectively (Fig. 1A and B). The most abundant alteration detected across all SF exposures was regions with upregulated 5hmC sites, namely hyper-differentially hydroxymethylated regions (hyperDhMRs), accounting for 93.3% of the total identified regions. Among the identified DhMRs, SF^110^ induced 100% (2/2) hyperDhMRs, SF^220^ induced 93.3% (1 188/1 273), SF^1100^ induced 93.1% (630/677), and SF^2200^ induced 100% (12/12). Further analysis of the genomic features associated with altered 5hmC levels following SF α-irradiation identified the gene body region (i.e. exons and introns) as the genomic location harbouring the highest percentage (52.1%) of hyperDhMRs, and the promoter site as the genomic region with the highest percentage of hypo-differentially hydroxymethylated regions (hypoDhMRs) with 40.2% (Fig. 1C).

**Figure 2:**
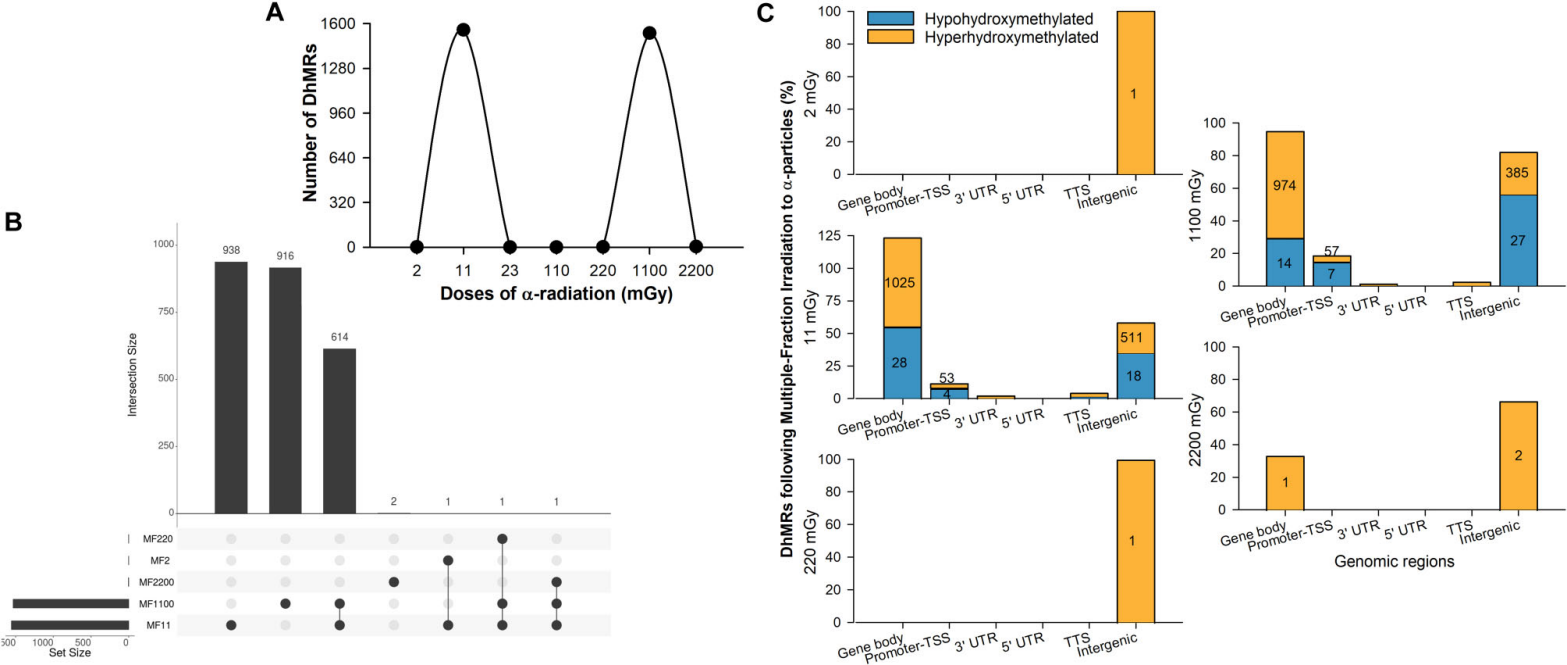
Differentially hydroxymethylated regions (DhMRs) in lung fibroblasts following multi-fraction (MF) α-irradiation. (A) Total number of DhMRs across seven americium-241 α-particle doses identified using the Bioconductor MEDIPS package. (B) UpSet plot displaying the number of identified DhMRs in the fibroblasts across the different MF α-irradiation doses. (C) Percentages of the genomic regions found to be hypo- and hyper-hydroxymethylated in each irradiated group. The integer inside each bar represents the number of DhMRs. Each irradiation group includes four biological replicates per dose, independently irradiated and assessed. TSS, transcription start site; TTS, transcription termination site; 3’ UTR, 3’ untranslated region; 5’ UTR, 5’ untranslated region.

Compared with SF exposures, fibroblast cells that underwent MF irradiation displayed a binomial-like dose-response relationship in the number of identified 5hmC modifications. The numbers of DhMRs computed from the differential analysis were 1; 1 555; 1; 1 532; and 3 for MF^2^, MF^11^, MF^220^, MF^1100^, and MF^2200^, respectively (Fig. 2A and B). Most (96.8%) of the alterations identified following MF α-irradiations were regions with increased 5hmC levels. Irradiation with MF^2^ induced 100% (1/1) hyperDhMRs, MF^11^ induced 96.7% (1 504/1 555), MF^220^ induced 100% (1/1), MF^1100^ induced 96.9% (1 484/1 532), and MF^2200^ induced 100% (3/3). Similar to the cells irradiated with SF exposures, the primary genomic region where hyperDhMRs were most abundant (66.8%) was the gene body, and the location where hypoDhMRs were most prevalent, with 45.5%, was the intergenic region (Fig. 2C).

### Alpha-irradiation modulates 5hmC levels in key DNAm enzymes

To establish a link between 5mC and 5hmC, we next assessed DNAhm levels in genes encoding the enzymes responsible for DNAm and demethylation. Our data showed hyperDhMRs in the body region of the *TET3* gene in the cells α-irradiated to SF^220^. We also detected a significant 5hmC increase in the body region of the DNA methyltransferase 3A (*DNMT3A*) gene in the DNA of the fibroblast cells irradiated with MF^11^. These results suggest that SF and MF α-radiation may modify the methylome and hydroxymethylome landscape of lung fibroblasts through 5hmC alterations in key enzymes involved in DNAm and demethylation pathways.

### MF irradiations, in contrast to SF, disrupt pathway-based biomarkers of radiation through 5hmC alterations in fibroblasts

The potential biological effects of each exposure regimen on the hydroxymethylome of the irradiated fibroblasts were then assessed. To focus on the specific and most prominent epigenetic responses within each irradiation regimen, we selected the doses that yielded the highest numbers of DhMRs for each regimen. Genes harbouring DhMRs in their promoter or gene body regions that were shared across the selected doses were retained for downstream functional analysis using the software QIAGEN Ingenuity Pathway Analysis (IPA) [30]. A combined analysis of promoter and gene body regions was performed, as hydroxymethylation at both sites has been associated with similar functional outcomes, namely a positive correlation with gene expression, thereby enabling a more comprehensive representation of gene activity at the pathway level. Based on the DhMR analysis, SF^220^ and SF^1100^ were the two SF doses that generated the highest numbers of DhMRs, while MF^11^ and MF^1100^ induced the highest numbers within the MF regimen.

**Figure 3:**
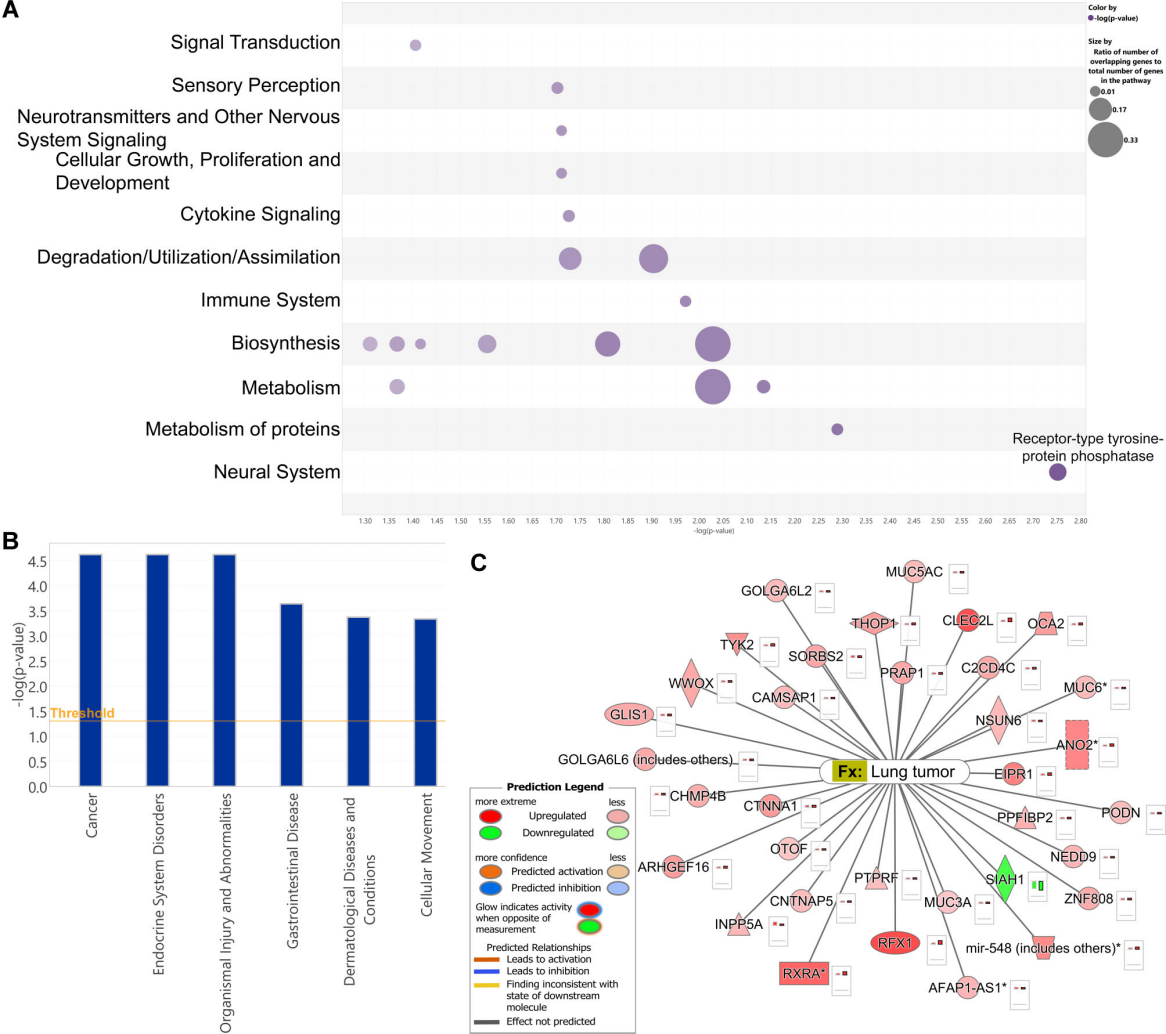
Biological pathways enrichment analysis of genes harbouring differentially hydroxymethylated regions (DhMRs) in promoters and gene body regions following single-fraction (SF) α-irradiation. (A) Enriched canonical pathways (*P*-value < 0.05). (B) High-level functional category of the most enriched diseases and functions ranked using p-values. (C) Selected diseases and functions from the SF list. The DNA hydroxymethylation status displayed in panel C is from SF^1100^. Panels A–C were generated using the genes commonly identified in SF^220^ and SF^1100^. The networks and functional analyses were generated using QIAGEN IPA (QIAGEN Inc., https://digitalinsights.qiagen.com/IPA). Fx: diseases and functions.

Functional analysis with the 103 commonly identified genes from the SF list yielded 19 enriched canonical pathways with *P*-values < 0.05. Most of these pathways were related to the categorical function of biosynthesis and metabolism (Fig. 3A). The complex receptor-type tyrosine-protein phosphatases were the most significant canonical pathway (*P*-value = 1.8 × 10^−3^). This type of receptor plays a crucial role in cell signaling regulation, including cell growth, proliferation, apoptosis, survival, invasion, and migration [31]. Consequently, dysregulation of the receptor-type tyrosine-protein phosphatase has been associated with carcinogenesis. Two genes, *PPFIBP2* and *PTPRF*, from the twenty genes involved in the receptor-type tyrosine-protein phosphatases pathway, were identified in our SF common list by IPA. Both genes harbour hyperDhMRs in the intron region in both the SF^220^ and SF^1100^ datasets, suggesting a potential association with increased expression levels and higher activity.

We also performed downstream analysis using the same SF gene list to predict diseases linked to the molecular changes observed in our common SF dataset. Cancer, with 71 associated genes from our list, was identified by IPA as the category with the most significant *P*-values (range 4.7 × 10^−2^–2.4 × 10^−5^) (Fig. 3B). Among the diseases and functions identified within this category, lung tumour was found to be significantly enriched (*p*-value = 1.3 × 10^−3^) with 34 genes (Fig. 3C). Lung tumour-related pathways are predicted to be activated in the SF^1100^-treated fibroblasts.

**Figure 4:**
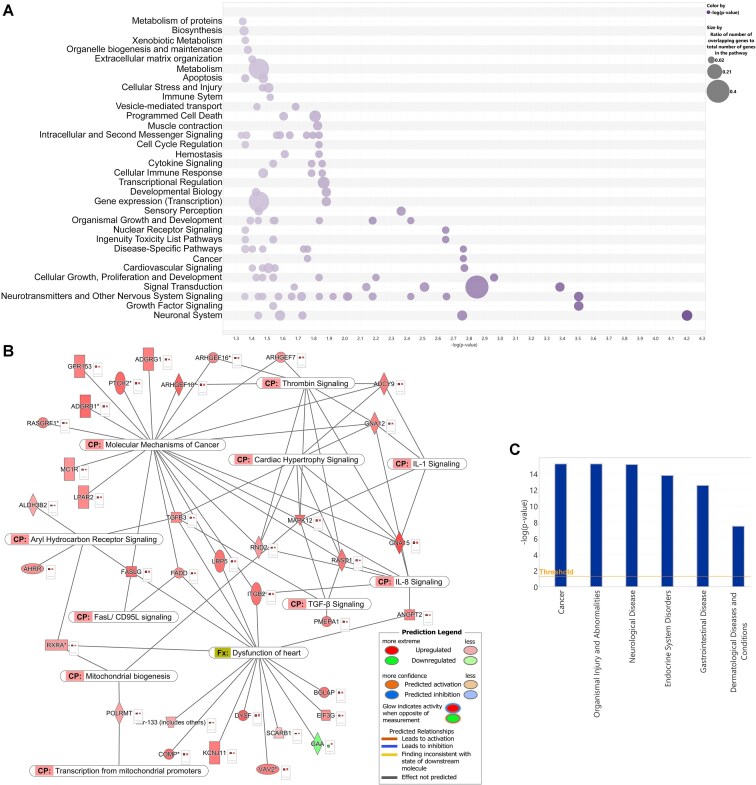
Biological pathways enrichment analysis of genes harbouring differentially hydroxymethylated regions (DhMRs) in promoters and gene body regions following multi-fraction (MF) α-irradiation. (A) Enriched canonical pathways (*P*-value < 0.05). (B) Selected canonical pathways, and diseases and functions from the MF list. The DNA hydroxymethylation status displayed in panel B is from MF^11^. (C) High-level functional category of the most enriched diseases and functions ranked using *P*-values. Panels A–C were generated using the genes commonly identified in MF^11^ and MF^1100^. The networks and functional analyses were generated using QIAGEN IPA (QIAGEN Inc., https://digitalinsights.qiagen.com/IPA). CP: canonical pathways; Fx: diseases and functions.

Comparison between MF^11^ and MF^1100^ resulted in 379 genes commonly harbouring DhMRs within their promoter or gene body region. Functional analysis of these 379 genes from the MF list uncovered 74 canonical pathways with *P*-values < 0.05. Most of these pathways were associated with categories involving cellular functions, including signal transduction, cellular growth, proliferation and development, cell cycle regulation, intracellular and second messenger signaling, programmed cell death, and apoptosis, in addition to disease-specific canonical pathways (Fig. 4A). Interestingly, these canonical pathways generated with the common MF gene list are highly correlated with processes underlying the cellular response to IR. The canonical pathways and genes from the MF list involved in these cellular processes are displayed in Fig. 4B. The FasL/CD95L signaling pathway is an apoptotic process that is activated by p53 in response to irreparable DNA damage triggered by IR [32]. Inflammatory response-related pathways are also activated by exposure to radiation. Some of these inflammatory processes enriched in our MF list were thrombin signaling [33], IL-8 signaling [34], and IL-1 signaling [35]. The two pathways, thrombin and IL-8 signaling, from our MF^11^ and MF^1100^ datasets were predicted by IPA to be activated, which agrees with known cellular responses to IR. Our datasets did not have sufficient gene targets for IPA to statistically predict the activation state of IL-1 signaling (i.e. increased or decreased). Other important pathways instigated by α-irradiation with MF^11^ and MF^1100^ were TGF-β signaling and aryl hydrocarbon receptor signaling. The TGF-β signaling plays an important role in the preservation of organismal integrity [36], whereas the aryl hydrocarbon receptor signaling is associated with cell proliferation, differentiation, and apoptosis [37].

Cellular responses to IR rely greatly on the role of mitochondria in providing genomic stability, mediating adaptive responses, and supporting cellular energy demands through mitochondrial respiratory pathways to maintain cellular integrity and functions [38]. In our MF list, we observed two mitochondrial-related canonical pathways significantly affected by MF radiation, namely transcription from mitochondrial promoters and mitochondrial biogenesis (Fig. 4B). A key catalytic component of the transcription from the mitochondrial promoters’ pathway is the mitochondrial DNA-directed RNA polymerase encoded by the mitochondrial RNA polymerase (*POLRMT*) gene. In both our MF datasets, this gene harbours hyperDhMRs within its gene body region, suggesting a potential increase in its expression levels. Mitochondrial biogenesis is an essential cellular process involved in the response to external insults, including IR. It provides the necessary energy to maintain cell homeostasis [39]. Based on the 5hmC levels of the genes involved in mitochondrial biogenesis, IPA predicted this pathway to be activated in our MF^1100^ dataset, whereas the MF^11^ exposures did not have sufficient gene targets for the prediction to be significant.

The genes from the MF list were also involved in disease-specific canonical pathways, including molecular mechanisms of cancer and cardiac hypertrophy signaling, two diseases highly correlated with exposure to IR. These pathways were predicted to be activated in both the MF^11^ and MF^1100^ datasets.

Pathway analysis for the identification of diseases and disorders involving the MF dataset resulted in cancer as the most enriched disorder, followed by organismal injury and abnormalities and neurological diseases (Fig. 4C). Delving further into each group, we found that neurotoxicity was the primary theme among these three categories. For instance, diseases including grade 1–4 astrocytoma and brain tumour with *P*-values of 4.5 × 10^−15^ and 1.5 × 10^−14^, respectively, were among the top eight disorders under cancer, organismal injury and abnormalities, and neurological diseases. Although seldom studied [40], neurotoxic effects among children and adolescents have been strongly associated with α-particle exposures, especially ^222^Rn and thoron gases [41, 42].

### DNAhm profile of aging-associated genes in the DNA of fibroblasts irradiated to SF and MF α-particles

To evaluate the DNAhm profile of aging-associated genes in the lung fibroblasts following SF and MF α-irradiation, we used the gene lists derived from two well-established epigenetic DNAm clock algorithms, namely Horvath’s epigenetic aging clock [43] and Levine and colleagues’ biological age calculator, known as PhenoAge [44]. These aging clocks were generated from selected genes that undergo DNAm changes consistently with aging, regardless of the cell or tissue type [26]. To identify DNAhm biomarkers of aging, we compared our DhMRs-associated genes obtained from the hMeDIP-seq experiment with the gene lists from Horvath’s epigenetic aging clock [43] and the PhenoAge clock [44]. It is worth noting that these comparisons are based on gene-level annotations of epigenetic clock CpG sites to genes associated with DhMRs in our dataset. Moreover, DNAm clocks are derived from 5mC data at specific CpG sites, whereas our analysis examines 5hmC. Accordingly, these comparisons should be interpreted with caution, as they are intended to capture gene-level relationships associated with the epigenetic regulation of aging, rather than to provide a direct analysis of epigenetic clock algorithms.

**Figure 5:**
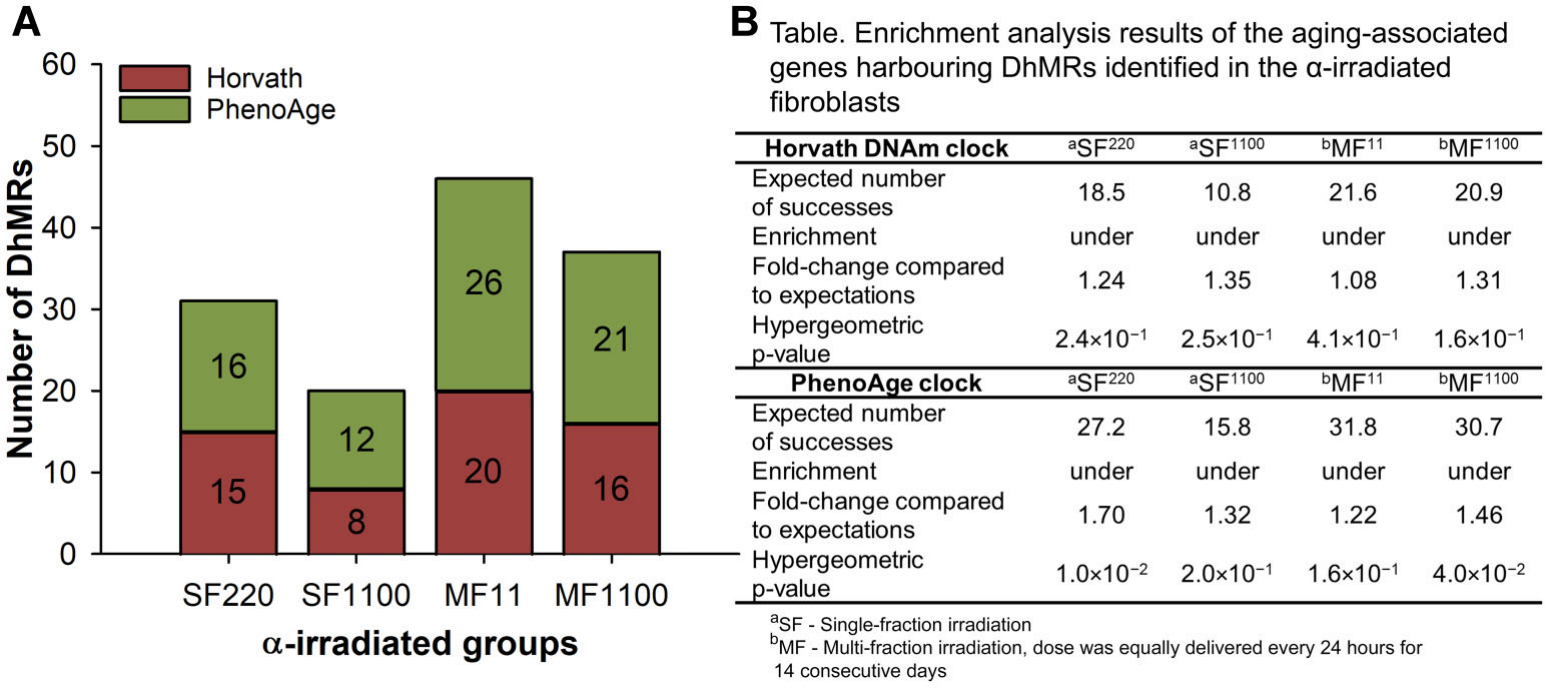
Aging-associated DNA hydroxymethylation events identified in the α-irradiated lung fibroblasts. These events are based on the DNA methylation (DNAm) clock from Horvath [43] and on the PhenoAge DNAm clock from Levine et al. [44]. (A) Number of differentially hydroxymethylated regions (DhMRs) from the α-irradiated fibroblasts annotated to genes using HOMER and identified as associated with aging, as established by the two selected DNAm clocks. (B) Table displaying the enrichment analysis results of the aging-associated genes whose 5hmC levels were altered in the α-irradiated fibroblasts, computed using the cumulative distribution function of the hypergeometric equation from [45].

Analysis of the SF-irradiated fibroblasts revealed 5hmC alterations in 4.4% of cellular aging-associated genes relative to Horvath’s DNAm clock and 3.2% relative to the PhenoAge clock’s genes following SF^220^ irradiations (Fig. 5A). The SF^1100^ affected 2.3% and 2.4% of the aging-associated genes in the α-irradiated fibroblasts relative to Horvath’s and PhenoAge DNAm clock’s genes, respectively (Fig. 5A). In the MF exposure groups, irradiations with MF^11^ and MF^1100^ triggered 5hmC changes in 5.8% and 4.7% of aging-related genes, respectively, relative to those from Horvath’s DNAm clock. Similarly, compared to the genes from the PhenoAge DNAm clock, MF^11^ and MF^1100^ modified the DNAhm profile of 5.1% and 4.2% of cellular aging-associated genes, respectively (Fig. 5A). These findings suggest that the MF regimen may exert a greater impact on the biological age of the α-irradiated cells, as irradiation protocols using the MF exposure regimen induced 5hmC alterations in a larger number of aging-associated genes compared with SF exposures. To corroborate this observation, we performed an enrichment analysis using the cumulative distribution function of the hypergeometric equation described by Graeber [45]. The results from this test confirmed that the MF irradiations affected the hydroxymethylome of a greater number of aging-related genes relative to SF exposures (Fig. 5B).

### Potential mitochondrial dysfunction resulting from 5hmC alterations in nuclear-encoded mitochondrial genes following SF and MF α-irradiation to fibroblasts

In our dataset, we did not identify any 5hmC alterations in the mtDNA. However, the hydroxymethylome profile of nuclear-encoded mitochondrial genes was found to be affected by α-particle radiation. These genes encoding mitochondrial proteins are located in the nucleus and represent ∼8.3% (∼1650 in 20 000) of the total number of nuclear genes [46, 47]. These nucleus-encoded genes are translocated to the mitochondria using specialized import systems and are required for the proper functioning of the mitochondria.

**Figure 6:**
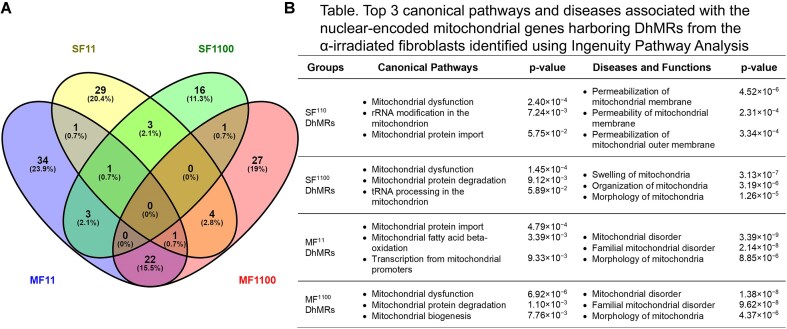
Nuclear-encoded mitochondrial genes identified following single-fraction (SF) and multi-fraction (MF) α-irradiation of fibroblasts. (A) Venn diagram exhibiting the number of nuclear-encoded mitochondrial genes whose differentially hydroxymethylated regions (DhMRs) were induced by SF and MF α-irradiation (Venn diagrams created using Oliveros [78]). (B) Table displaying the top three canonical pathways and diseases using QIAGEN IPA associated with the nuclear-encoded mitochondrial genes harbouring DhMRs.

Using the 1655 nuclear-encoded mitochondrial gene list by Elsadany et al. [47], we identified 39; 24; 60; and 55 of these genes harbouring DhMRs in the fibroblasts α-irradiated with SF^110^, SF^1100^, MF^11^, and MF^1100^, respectively (Fig. 6A). To investigate the biological pathways of these nuclear-encoded mitochondrial-affected genes potentially disrupted by the SF- and MF-irradiations, we then performed enrichment analysis using IPA. The analysis revealed that mitochondrial dysfunction was one of the three most significantly mitochondrial-enriched canonical pathways across SF^110^, SF^1100^ and MF^1100^ irradiated groups (Fig. 6B). Furthermore, functional analysis indicated that these genes were commonly linked to diseases and abnormalities of the mitochondrion, including mitochondrial disorders, and morphology of mitochondria, such as their length, mass, shape, and size.

### MF α-irradiations increase mtDNA copy number per cell

The oxidative stress and other non-targeted effects induced by exposure to IR may trigger both nuclear DNA and mtDNA damage, leading to genomic instability [29]. The extent of this damage is highly dependent on the dose, dose rate, exposure length, and radiation quality. Despite the mtDNA repair capabilities, the mitochondria are less efficient at mending oxidative damage compared with their counterpart, the nuclear DNA [48]. Given the pivotal role of mitochondria in regulating epigenetic mechanisms and the capacity of IR to potentially impair mitochondrial function through mtDNA damage [29], we examined mtDNA aberrations, including the quantification of common IR-induced deletions as well as mtDNA copy number.

**Figure 7:**
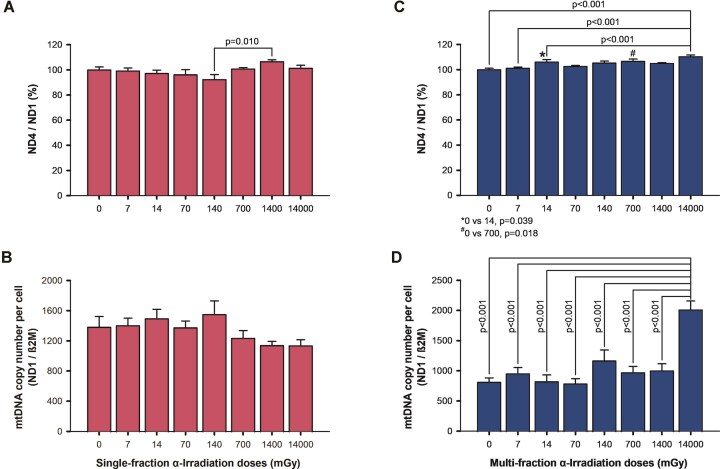
Quantification of mitochondrial DNA (mtDNA) damage induced by α-irradiation to lung fibroblasts. Percentage of deletions and mutations in the *ND4* gene (A), and mtDNA copy number per cell (B) in the second set of SF α-irradiated fibroblasts. Percentage of deletions and mutations in the *ND4* gene (C), and mtDNA copy number per cell (D) in the second set of MF α-irradiated fibroblasts. Data are presented as mean ± SEM and analyzed by one-way ANOVA, with a sample size of eight biological replicates per dose.

These mtDNA endpoints were assessed using two different sets of α-irradiated cells. The first set consisted of the same irradiated cells used for our epigenetic endpoints. Although a sample size of four per dose was appropriate for the hMeDIP experiment, it was not statistically robust to quantify mtDNA aberrations. Therefore, a second set of α-irradiations following the same experimental procedure was conducted using a dose range between 0 and 14 000 mGy and a sample size of eight per dose. For this second set of irradiations, slightly different doses were selected to achieve a broader distribution and better capture the dose-response relationship, which may be masked when using closely spaced doses, particularly for mtDNA damage. The α-irradiations were also performed using the SF and MF delivery methods. The results of these two sets of irradiations are shown in Supplementary file1, Fig. 1 for the first set of α-irradiations and Fig. 7 for the second set of exposures. Since the second set of irradiations was more statistically robust, only their effects are described in this section.

Radiation-induced mtDNA damage [38], normal aging processes, and age-related diseases [49] have all been associated with common deletions in the major arc of the mtDNA. Therefore, using a droplet digital polymerase chain reaction (ddPCR) system, we assessed the deletion ratio of a mtDNA region located within this arc, specifically a site within the NADH dehydrogenase subunit 4 (*ND4*) gene. As a reference for the total amount of mtDNA copies, hereinafter referred to as wild-type mtDNA, we used a region within the NADH dehydrogenase subunit 1 (*ND1*) gene from the mtDNA minor arc, a site rarely deleted [49]. The deletion ratio is represented by:

Deletion ratio (%) = (wild-type *ND1* mtDNA—non-mutated *ND4* mtDNA)/(wild-type *ND1* mtDNA) × 100, where wild-type *ND1* mtDNA represents total mtDNA.

However, for better visualization, the graphs from Supplementary file1, Figs. 1A and C and 7A and C illustrate the percentage of non-mutated *ND4* mtDNA copies relative to wild-type *ND1* mtDNA copies.

An increase in mtDNA copy number following exposure to IR has been reported both *in vitro* and *in vivo* [50]. Therefore, to determine the mtDNA copy number per cell, we used the ratio between the wild-type *ND1* mtDNA gene and a nuclear target, specifically a site within the beta-2-microglobulin (*β2M*) gene, as suggested by Phillips et al. [49], due to its low variability and single copy.

A one-way ANOVA was conducted to examine the mtDNA deletion ratio and the mtDNA copy number following SF and MF α-irradiations. For the SF irradiation, the second set of cells showed a significant difference in the deletion ratio of *ND4* across radiation doses [*F*(_7,55_) = 2.414, *P* = 0.031]. In particular, a ∼14% significant increase in the deletion ratio was detected in the SF^140^ irradiated cells relative to the SF^1400^ irradiated cells (Fig. 7A). No effects were observed in the mtDNA copy number of the irradiated cells (Fig. 7B).

The MF irradiations induced a significant change in the deletion ratio of the *ND4* gene in the second set of irradiated cells across doses [*F*(_7,53_) = 5.987, *P* < 0.001] (Fig. 7B). Interestingly, the mtDNA copy number of this set of cells was also found to be significantly altered across doses [*F*(_7,52_) = 12.290, *P* < 0.001]. A two- to three-fold increase in mtDNA copy number was detected in cells irradiated to MF^14000^ compared with cells irradiated to MF^0^, MF^7^, MF^14^, MF^70^, MF^140^, MF^700^, and MF^1400^ (Fig. 7D).

## Discussion

In our previous study [26], we reported on the effects of both human-relevant doses of α-radiation and exposure regimen, namely SF and MF exposures, on the methylome profile of a normal human embryonic lung fibroblast cell line. Here, we used the same α-irradiated cells to investigate alterations in DNAhm, an understudied epigenetic mark and the most abundant oxidative derivative of DNAm known to play a pivotal role in the regulation of gene expression.

Prior studies assessing the hydroxymethylome in tissues other than the brain are scarce. In addition, to our knowledge, no studies have investigated the effects of high linear energy transfer (a measure of ionizing track density) or compared the changes induced by a pro-oxidant after different exposure regimens. This study aimed to delve into the significant 5hmC alterations following α-irradiation of lung cells using two human-relevant delivery methods, namely SF and MF exposures, designed to model acute and repetitive irradiation conditions. Incidents of α-particle exposure as a SF are infrequent, yet highly possible, and are primarily from the occurrence of nuclear accidents. On the other hand, human exposure to MF α-radiation is ubiquitous in the context of residential and/or occupational ^222^Rn exposures [5, 51], and to a lesser extent in radiotherapy.

A notable contrast between the effects of SF and MF irradiations on the hydroxymethylome lies in their dose–response relationships. Although the total doses between the two exposure regimens were kept consistent, the MF irradiation induced the greatest number of 5hmC alterations (>650 DhMRs) in the low dose (MF^11^) and high dose (MF^1100^) examined compared with the SF, where changes were observed only at high doses (SF^220^ and SF^1100^). A difference in dose–response was also detected in our previous study on the methylome landscape of the same α-irradiated lung cells [26], where we identified a large number of 5mC changes at a medium SF dose (SF^110^) and a dose-dependent increase in 5mC alterations for the MF irradiations. Differences in the molecular outcome between single and fractionated delivery of IR have also been reported in clinical studies in which optimal radiation exposure was sought for patients with cancer, especially non-small cell lung cancer [52–54]. To date, conflicting results remain regarding the effects of SF and MF radiation on therapeutic outcomes and survival rates of treated patients, with mechanisms underlying these observable differences not yet established. It is unclear why dose–response differences in 5hmC were exhibited in the SF and MF α-irradiations. These observable differences could be influenced by bystander effects [55], reoxygenation of irradiated cells, and/or allotted time for DNA repair [54].

In the endeavour to better understand the role of 5hmC in transcriptional activity, a couple of studies have examined the relationship between 5hmC distribution and global gene expression profile in different tissues. These studies showed 5hmC exhibiting specific genomic localization, particularly in gene body regions, which has been demonstrated to be a better correlation with enhanced expression levels compared with 5mC [14, 16, 19]. Little is known about the 5hmC landscape in lung fibroblasts following IR. However, our results showing high 5hmC levels in the body region of genes identified following SF and MF α-irradiations agree with the typical genomic accumulation of 5hmC. Notably, to our knowledge, this study is among the first to demonstrate that gene body regions in lung fibroblasts exhibit the highest levels of 5hmC enrichment following SF and MF α-irradiation, highlighting these genomic features as particularly susceptible to radiation-induced alterations in DNAhm, which is often linked to transcriptionally active genes.

In our previous study [26], we reported on the DNAm profiles of lung fibroblasts α-irradiated using either the SF or MF regimen, the same irradiated cells used in the present study. The SF α-irradiation primarily decreased 5mC levels in the irradiated cells, whereas elevated 5mC sites were mainly observed following the MF irradiations compared with the control cells. Active demethylation is performed by the TET family of enzymes, TET1, TET2, and TET3, which oxidize 5mC into 5hmC. Interestingly, in this study, we identified hyperDhMRs in the body region of the *TET3* gene in the cells irradiated with SF^220^. Increased 5hmC levels in gene body regions have been strongly associated with increased gene expression levels. Therefore, hyperDhMRs in the body region of *TET3* may suggest a potential enhancement in its expression levels and, consequently, its demethylation activity in the SF^220^-irradiated cells. However, this hypothesis requires further experimental validation. Although quantitative PCR analysis of *TET3* was considered to assess its transcription levels, it was not feasible due to limited sample availability. Notably, the findings from the current study are consistent with our previous results obtained from the same SF α-irradiated lung cells. In our earlier work, we reported reduced 5mC levels identified within the gene body region [26], whereas the present study identified increased 5hmC levels within the same genomic region. In support of these data, TET3 has been linked with the modulation of the DNA damage response [56], a process induced by DNA-damaging agents, including IR [32].

Another key enzyme involved in DNAm and whose gene was identified in our dataset is DNMT3A. HyperDhMRs were detected in the body region of the *DNMT3A* gene in the DNA of the fibroblast cells irradiated to MF^11^. DNMTs are enzymes that regulate DNAm by catalyzing the transfer of a methyl group from S-adenosyl-l-methionine to carbon-5 of cytosines in the CpG dinucleotide, forming 5mC [57]. Three families of DNMT enzymes, DNMT1, DNMT2, and DNMT3, have been identified in mammals. The members of the DNMT3s play a critical role in *de novo* DNA methyltransferase activity and, to some extent, in the maintenance of methylation patterns of the cell [58]. More specifically, DNMT3A is highly involved during early embryonic development, and it has also been associated with tumour-suppression activity in lung tumour progression [59]. Therefore, elevated 5hmC levels at the body region of the *DNMT3A* gene may suggest an increase in its expression levels and could indicate *de novo* methylation activity, which correlates with the high 5mC levels identified in our MF^11^ irradiated cells published in our previous study [26]. It is also worth noting that the methylome of the fibroblasts exhibited a defensive response against carcinogenesis to the MF α-irradiation, which is supported by the tumour-suppression activity role of DNMT3A.

The most prominent long-lasting deleterious outcome of exposure to low-ionizing-density gamma radiation is inflammation [20], and it has been well-documented among atomic bomb survivors [60–62]. Surprisingly, only the MF high-ionizing-density α-radiation, as opposed to the SF, induced 5hmC alterations in genes associated with inflammatory activity and other biomarkers of radiation-related pathways. Since inflammatory processes are triggered by both mitochondrial dysfunction and DNA damage response [63], we posit that the MF α-irradiation results in a greater extent of nuclear and mitochondrial DNA damage compared with the other exposure regimen, SF irradiation. The proposed effect is probably due to the daily irradiations under the MF regimen that limit the time available for effective cellular repair mechanisms. This is supported by the observed 5hmC enrichment in our MF-irradiated cells, consistent with its known active localization to DNA damage sites to promote genomic stability [64]. The hypothesis is further reinforced by the effects detected in the mitochondria of the MF-irradiated cells. One of these effects is the identification of hyperDhMRs in the body regions of the genes involved in mitochondrial biogenesis, which was predicted to be significantly activated in the MF-irradiated cells. To cope with IR-induced stress on energy metabolism, the cell enhances the number of super complexes in the electron transport chain, and also activates mitochondrial biogenesis [65, 66], a process involving mitochondrial elongation and/or self-replication, that ultimately leads to an increase in mtDNA copy number. A growth in mtDNA copy number was observed in the highest dose (MF^14000^) of our second irradiated set of MF exposures relative to the control and other examined doses. Although we did not detect any meaningful deletions in our SF- or MF-irradiated cells, it is worth noting that in most cells, the mtDNA mutations are heteroplasmic with the co-existence of both wild-type and mutant mtDNA [67]. Consequently, accurate quantification of heteroplasmic mtDNA mutations may present considerable challenges as the mtDNA mutations exhibit significant variability among the cell’s numerous mitochondria.

In conjunction with the previous findings on the mtDNA, some of the hyperDhMRs identified in the MF irradiations were located within genes associated with other biological pathways involved in the response to IR. One of these processes was the FasL/CD95L signaling, which plays a critical role in identifying and eliminating radiation-induced damaged cells [32, 68]. Another pathway involved in the radiation response and identified through pathway analysis in our MF-irradiated cells was TGF-β signaling. This pathway is known to play a role in tumour progression [69]. The extent of the involvement of TGF-β signaling highly depends on the isoform that is activated [70]. In our study, the isoform affected by the alteration of 5hmC levels was the *TGF-β3* gene, shown to have mitigation capabilities of radiation-induced toxicity in healthy tissue. Aryl hydrocarbon receptor signaling is another process altered by our MF exposures through changes in the 5hmC levels of genes involved in this pathway. This signaling is activated as part of the cellular response to oxidative stress [71]. Taken together, these findings suggest that MF pro-oxidant α-irradiations lead to greater nuclear and mitochondrial DNA damage compared with SF exposures, effects manifested by adaptive remedial responses in both mtDNA copy number and 5hmC levels in genes involved in key processes of the DNA damage response.

Given that 5hmC dynamics in human cellular aging are largely unknown [15], a primary focus of this study was to examine the 5hmC profile of established aging genes in lung cells *in vitro* irradiated with SF and MF α-particles. Using aging-associated genes from two well-established DNAm-based age predictor models, we found that MF irradiations induced alterations in DNAhm patterns across a larger set of genes associated with DNAm biomarkers of aging compared with SF exposures. Interestingly, the low dose of MF^11^ disrupted 5hmC sites in more aging-related genes relative to the high dose of MF^1100^. Since 5hmC is catalyzed from 5mC, the observed dose-effect pattern of MF-induced 5hmC alterations in aging-related genes correlates with the dose-dependent increase trend detected in our 5mC study [26]. Considering the large number of aging-associated genes affected by aberrations in 5mC and 5hmC levels, these findings suggest that MF α-irradiations, in contrast to SF, may exert a more profound influence on age-related processes, thereby potentially affecting biological age, through dysregulation of both 5mC and 5hmC profiles. Although the 5hmC alterations induced by MF^11^ may indicate dysregulation of aging-associated pathways, they may also result from an adaptive response to the applied stressor, as lower-dose exposures are known to promote adaptive cellular responses [72]. Distinguishing between these two mechanisms would require 5hmC assessment at later time points following irradiation, which was beyond the scope of this study but should be explored in future work. Notably, while we observed overlap between genes associated with DhMRs in our SF- and MF-irradiated cells and those linked to CpG sites from established epigenetic clocks, this analysis should be interpreted in light of important methodological constraints. DNAm clocks are derived from methylation states at specific CpG sites, whereas our analysis was conducted at the gene level; thus, mapping CpGs to genes requires an additional level of interpretation and does not preserve site-specific information. In addition, epigenetic clocks are based on 5mC patterns, while this study examines the 5hmC landscape, which may differ in functional relevance. Furthermore, CpG sites included in epigenetic clocks are selected collectively for their predictive value for biological age, reflecting multivariate methylation patterns rather than changes at individual loci. Despite these limitations, our analysis provides exploratory insight into the extent to which epigenetic alterations, specifically 5hmC, induced by SF and MF α-irradiations align with aging-associated signatures captured by DNAm clocks, suggesting a potential influence on biological age.

A limitation in our study is the inability to directly and accurately compare the 5hmC patterns instigated by the two assessed irradiation regimens, namely SF and MF exposures. The difference in time between the initial exposure and harvest time for the SF and MF irradiation conditions may be a critical factor influencing these comparisons. The cells from the SF regimen were collected 1 h post-irradiation. In contrast, the cells from the MF exposures were harvested 1 h following the last fraction of α-irradiation, or 14 days + 1 h from the first fraction. This difference in sampling time points limits direct comparison between conditions and underscores the need for further validation of the SF regimen. Future studies incorporating parallel-time course sampling following SF exposure, including alignment with MF irradiation time points, will be important to strengthen comparative interpretation. Additionally, the effects observed following MF irradiation may reflect incomplete cellular recovery, as repeated exposure could prolong the return to baseline conditions. Accordingly, a time-course study incorporating longer post-irradiation intervals would help better assess these effects. Another shortcoming of the present research is the use of the WI-38 cell line, which is derived from a 3-month-old female. Careful consideration must be given when extrapolating these results to adult tissue, as embryonic and adult cells do not share similar hydroxymethylome profiling. Consequently, the response of embryonic lung cells to radiation may differ from that of established adult cells. However, it is important to note that environmental exposures to pollutants or ^222^Rn are generally across all developmental stages, unlike smokers’ exposure, mainly in adulthood to cigarette smoke.

Overall, our work elucidates the contrasting landscape of an important and understudied epigenetic modification, 5hmC, between two α-irradiation exposure regimens, SF and MF, to which humans are routinely subjected. Following genome-wide profiling of 5hmC on the DNA of 32 SF and 32 MF α-irradiated lung fibroblast samples, we identified a dose-dependent increase in DNAhm levels regardless of the exposure regimen (SF or MF irradiation). Functional analysis of the genes affected by the increased 5hmC levels revealed a profound disruption of pathway-based radiation biomarkers following MF irradiations compared with SF exposures. Interestingly, we also detected 5hmC alterations in key genes encoding enzymes involved in DNAm and demethylation processes, which supports the findings from our previous study on the methylome [26]. Furthermore, although both the SF and MF α-irradiations altered the hydroxymethylome of aging-related genes, the MF regimen led to 5hmC changes in a greater number of these genes. At equivalent SF doses, the MF α-irradiation induced more mitochondrial damage, effects that were exhibited as alterations in the hydroxymethylome of nuclear-encoded mitochondrial genes and mtDNA copy number. Humans are repeatedly exposed to varying levels of ^222^Rn-derived α-particles as they occupy residential and non-residential buildings and are exposed to generally lower levels in outdoor environments. Hence, exposures occur over a wide range of doses and dose rates [73–75], and therefore conducting studies with a broad range of exposure characteristics is important to better understand the associated health effects.

## Materials and methods

### Cell culture and α-irradiation

Normal fibroblast cell cultures derived from female lung tissue (human WI-38) were maintained and irradiated as previously described [26]. In brief, cells were subjected to seven doses of α-particles, each irradiated using two exposure regimens, a SF (total dose delivered in one day) and a MF (total dose equally distributed into 14 fractions, with one fraction delivered every 24 h). A 14-day exposure period was selected to model repeated and prolonged conditions, similar to chronic residential ^222^Rn exposure, while remaining within the upper range for maintaining cellular integrity.

The α-irradiation total doses for the first set of cells were 2.0; 11; 23; 110; 220; 1 100; and 2 200 mGy. The irradiation per dose for each regimen was conducted in four replicates. Cells in both SF and MF exposure regimens were trypsinized and collected after the last irradiation following 1 h incubation at 37°C in a 5% CO_2_ humidified atmosphere [26].

The α-irradiation total doses for the second set of cells were 7.0; 14; 70; 140; 700; 1 400; and 14 000 mGy. The irradiation per dose for each regimen was conducted in eight replicates. Cells in both SF and MF exposure regimens were trypsinized and collected after the last irradiation following 24 h and 1 h incubation for the SF and MF regimens, respectively, at 37°C in a 5% CO_2_ humidified atmosphere [26].

The first set of irradiations was performed with a sample size of four replicates, as it provides sufficient statistical power for the hydroxymethylation analysis, exceeding the minimum requirements for robust detection. In contrast, this level of replication was insufficient for the mitochondrial endpoints, for which a total of eight replicates was required to ensure robust results.

### DNA extraction and hMeDIP-seq

Total DNA extraction from frozen cell pellets, library preparation, and hydroxymethyl immunoprecipitation (IP) were conducted as previously described in our methylated DNA immunoprecipitation coupled with sequencing (MeDIP-seq) experiment [26] with some modifications. In hMeDIP-seq, the resulting adaptor-ligated DNA underwent IP with a 5-hydroxymethylcytosine (5-hmC) monoclonal antibody (mouse) using an hMeDIP kit (Diagenode, US) as described in the manufacturer’s protocol, with the adjustment that the starting volume of the adaptor-ligated DNA for the IP incubation was 15 µl (i.e. the volume of water was adjusted).

The recovery and enrichment of the hydroxymethylated DNA fragments (immunoprecipitated samples) were assessed using the provided primer sets specific for the spike-in controls, including hydroxymethylated DNA, methylated DNA, and unmethylated DNA (Diagenode, US). The average recovery rate of hydroxymethylated DNA was found to be 88%, whereas the methylated DNA fragments had an average recovery rate of less than 0.2%, and that of unmethylated fragments was less than 0.1%. The enrichment of the libraries was calculated to be 8 620-fold. Sequencing was conducted using an Illumina NovaSeq 6000 S4 platform (Illumina) at Génome Québec (Montréal, Canada), which generated around 131—233 million raw reads per library.

### Sequencing data processing

Data were analyzed as previously described [26] with some modifications. The differential coverage for the identification of DhMRs between controls and irradiated cells was calculated using the edgeR function in MEDIPS (v1.40.0) [76] with a minimum of five reads with TMM normalization. Statistically significant methylation coverage results were selected using the false discovery rate criterion of < 0.05. Enrichment analysis was performed using QIAGEN IPA [30].

### Assessment of mtDNA endpoints

Total DNA previously extracted was used to quantify mtDNA deletions and copy number. The mtDNA endpoints from the first (64 samples) and second (128 samples) sets of SF and MF α-irradiations were quantified by ddPCR (Bio-Rad, CA). Assessment of the mtDNA deletion ratio induced by α-radiation was performed by quantifying the amplification of two strategically selected mitochondrial targets, namely *ND1* and *ND4*. To determine the mtDNA copy number, we used the ratio of the mitochondrial *ND1* and the nuclear target *β2M*. Sequences for the specific primers and probes for *ND1, ND4*, and *β2M* were previously reported by Xue et al. [77], Rygiel et al. [67], and Phillips et al. [49], respectively, and displayed in Table 1. A mislabeling error was detected in Xue et al. [77]; the authors used the incorrect primer and probe sequences for the specific gene name. The sequences provided for gene *ND6* are the ones for *ND1*, which have been corrected in this study. The reaction was conducted in a 20 μl PCR mix containing 1X ddPCR Supermix for probes (no dUTP) (Bio-Rad, CA), primers (0.25 μM for *ND1* and *ND4* and 22.5 μM for *β2M*) (Integrated DNA Technologies, US) and 0.25 μM probes (Applied Biosystems, US) with the following cycling profile: one cycle of 95°C for 10 min, 40 cycles of 94°C for 30 s, and 60°C for 1 min, and one cycle of 98°C for 10 min and 4°C for 10 min. Due to the high ratio of mtDNA to nuclear DNA, different DNA template dilutions were used for each target. The mtDNA copy number was calculated after correction for dilution factors by $\frac{{ND1\ \textit{copies}\ \times \ 2}}{{\beta 2M\ \textit{copies}}}$.

**Table 1:** Sequences of primers and probes for the assessment of mtDNA deletion ratio and copy number. The positions of the binding sites refer to accession number DQ217933.1 for the nuclear gene and NC_012 920 for the mtDNA. mt, mitochondrial; nc, nuclear.

Target (gene name)	Binding site	Sequence (5’–3’)	Reference
*ND1*	mt 3 318–3 341	F—CCCTAAAACCCGCCACATCT	
	mt 3 476–3 496	R—GAGCGATGGTGAGAGCTAAGGT	[77]
		P—FAM-TTCGCTGACGCCATAA-MGB	
*ND4*	mt 12 087–12 109	F—CCATTCTCCTCCTATCCCTCAAC	
	mt 12 140–12 170	R—CACAATCTGATGTTTTGGTTAAACTATATTT	[67]
		P—FAM-CCGACATCATTACCGGGTTTTCCTCTTG-MGB	
*β2M*	nc 6 526–6 552	F—GCTGGGTAGCTCTAAACAATGTATTCA	
	nc 6 593–6 620	R—CCATGTACTAACAAATGTCTAAAATGGT	[49]
		P—FAM-CAGCAGCCTATTCTGC-MGB	

### Statistical analyses for mtDNA endpoints

Statistical analyses were conducted using SigmaPlot v15.0 (Grafiti, US). For one-way ANOVAs, datasets were examined for normality and homogeneity of variance using the Shapiro–Wilk test and Brown–Forsythe test, respectively. Box–Cox transformations were applied when the data were not normally distributed. Alternatively, one-way ANOVA on ranks indicated in each graph, where applicable, was used when the distribution of the data was non-Gaussian even after undergoing transformations. Significance was set at *P* < 0.05. To compare significance within the groups, the analysis was followed by Tukey’s post hoc test.

## Supplementary Material

dvag020_Supplemental_File

## Data Availability

The hMeDIP-seq data from primary lung fibroblasts from control and irradiated to a wide range of doses of α-particles reported in this paper are deposited in Gene Expression Omnibus (GEO) database under accession number GSE284411(https://www.ncbi.nlm.nih.gov/geo/query/acc.cgi?acc=GSE284411).
