# Supplementary material for: Distinct DNA hydroxymethylation landscape and mitochondrial DNA rearrangement signatures induced by single- and multi-fraction alpha particle radiation in lung fibroblasts
Source: Environ Epigenet. 2026 Jul 23;12(1):dvag020. doi: 10.1093/eep/dvag020 (PMC13392570; doi:10.1093/eep/dvag020)
Supplement: dvag020_Supplemental_File [file dvag020_supplemental_file.docx]

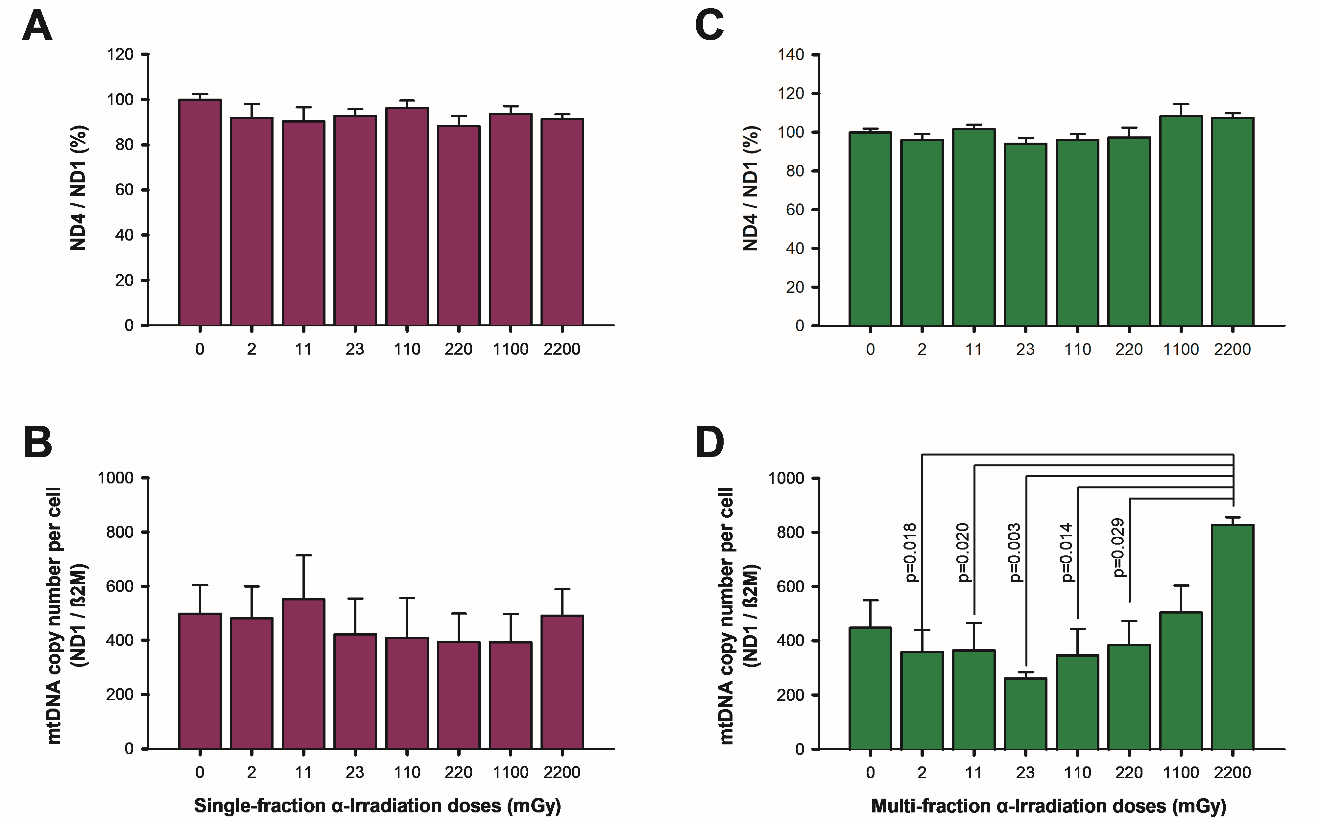


Figure S1. Quantification of mitochondrial DNA (mtDNA) damage induced by α-irradiation to lung fibroblasts. Percentage of deletions and mutations in the *ND4* gene (A), and mtDNA copy number per cell (B) in the second set of SF α-irradiated fibroblasts. Percentage of deletions and mutations in the *ND4* gene (C), and mtDNA copy number per cell (D) in the second set of MF α-irradiated fibroblasts. Data are presented as mean ± SEM and analyzed by one-way ANOVA (on ranks for B). Sample size of 4 biological replicates per dose.

Table S1. Number of differentially hydroxymethylated regions (DhMRs) in α-irradiated fibroblasts. The dose was delivered either as a single-fraction or equally distributed in 14 fractions (multi-fraction) with one fraction per day every 24 hours. The DhMRs were generated using the MEDIPS package. The adjusted p values were computed using the false discovery rate (FDR) method.

| **Total dose**  **(mGy)** | **Single-fraction Exposure** | | | | **Multi-fraction Exposure** | | | |
| --- | --- | --- | --- | --- | --- | --- | --- | --- |
|  | **Raw**  **p value**  **<0.05** | **Adjusted**  **p value**  **<0.1** | **Adjusted**  **p value**  **<0.05** | **Merged**  **FDR**  **<0.05** | **Raw**  **p value**  **<0.05** | **Adjusted**  **p value**  **<0.1** | **Adjusted**  **p value**  **<0.05** | **Merged**  **FDR**  **<0.05** |
| 2.0 | 683,472 | 1 | 0 | 0 | 635,694 | 1 | 1 | 1 |
| 11 | 666,103 | 0 | 0 | 0 | 798,891 | 4,575 | 2,866 | 1555 |
| 23 | 626,320 | 0 | 0 | 0 | 595,709 | 0 | 0 | 0 |
| 110 | 735,454 | 7 | 3 | 2 | 673,359 | 0 | 0 | 0 |
| 220 | 803,302 | 4,113 | 2,298 | 1,273 | 580,536 | 5 | 4 | 1 |
| 1,100 | 970,992 | 2,385 | 853 | 677 | 693,542 | 4,373 | 2,866 | 1,532 |
| 2,200 | 707,613 | 25 | 15 | 12 | 612,305 | 10 | 5 | 3 |
